# Visibiome: an efficient microbiome search engine based on a scalable, distributed architecture

**DOI:** 10.1186/s12859-017-1763-0

**Published:** 2017-07-24

**Authors:** Syafiq Kamarul Azman, Muhammad Zohaib Anwar, Andreas Henschel

**Affiliations:** 1 Department of Electrical Engineering and Computer Science, Masdar Institute of Science and Technology, Masdar City, Abu Dhabi, UAE; 2 Department of Environmental Science, Aarhus University, Frederiksborgvej 399, Roskilde, Denmark

**Keywords:** Microbiome, Microbial diversity, Search engine

## Abstract

**Background:**

Given the current influx of 16S rRNA profiles of microbiota samples, it is conceivable that large amounts of them eventually are available for search, comparison and contextualization with respect to novel samples. This process facilitates the identification of similar compositional features in microbiota elsewhere and therefore can help to understand driving factors for microbial community assembly.

**Results:**

We present Visibiome, a microbiome search engine that can perform exhaustive, phylogeny based similarity search and contextualization of user-provided samples against a comprehensive dataset of 16S rRNA profiles environments, while tackling several computational challenges. In order to scale to high demands, we developed a distributed system that combines web framework technology, task queueing and scheduling, cloud computing and a dedicated database server. To further ensure speed and efficiency, we have deployed Nearest Neighbor search algorithms, capable of sublinear searches in high-dimensional metric spaces in combination with an optimized Earth Mover Distance based implementation of weighted UniFrac. The search also incorporates pairwise (adaptive) rarefaction and optionally, 16S rRNA copy number correction. The result of a query microbiome sample is the contextualization against a comprehensive database of microbiome samples from a diverse range of environments, visualized through a rich set of interactive figures and diagrams, including barchart-based compositional comparisons and ranking of the closest matches in the database.

**Conclusions:**

Visibiome is a convenient, scalable and efficient framework to search microbiomes against a comprehensive database of environmental samples. The search engine leverages a popular but computationally expensive, phylogeny based distance metric, while providing numerous advantages over the current state of the art tool.

**Electronic supplementary material:**

The online version of this article (doi:10.1186/s12859-017-1763-0) contains supplementary material, which is available to authorized users.

## Background

Similarity search of microbial community profiles against a comprehensive microbiome database can unravel surprising results. For example, [1] reports that samples taken from 2.5 km below the deep-sea surface are closer to organotrophic forest soils in terms of microbial composition than to samples of shallower depths from the same study. This similarity is attributed to the abundance of methanogens. Like in the above-mentioned case, to understand the environmental factors that govern microbial community assembly for a particular sample at hand, it is desirable to find the most similar microbial communities that have been investigated, sequenced and deposited by other researchers. The subsequent analysis of commonalities with respect to their isolation source, description and environmental factors that have led to the observed taxonomic composition of community constituents can unravel the underlying ecological mechanisms and functionality aspects. Such comparison faces three main requirements: (i) the consistent deposition of microbial community profiles in suitable databases, including standardized metadata, (ii) the availability of tools that analyze microbial communities and (iii) the possibility to query against a comprehensive database of diverse samples.

The former two problems have been readily addressed. Thanks to advances in metagenomics, environmental sampling of microbial communities using Next Generation Sequencing and multiplexing, large amounts of descriptive genetic data are accumulated, particularly 16S rRNA profiles of microbial communities. Moreover, recent years have seen a dramatic increase in microbiome research, which is in part due to the fact that the role of the microbiome is recognized in a wider range of diseases but also environmental processes. Notable trailblazing efforts are the Human Microbiome Project [2] and the Earth Microbiome Project [3]. However, few problems remain and reflect on the quality of solutions for the third problem. For example, the importance of metadata annotation has been emphasized in [4], but the complete and consistent implementation of the developed standards is still in a nascent state. As a result, microbiome search engines can currently not be equipped with search criteria such as pH, salinity, isolation source or temperature. The third problem, to query a user provided sample against a large, comprehensive dataset has not been tackled, except for very few approaches [5]. The task of comparing microbial community profiles is computationally expensive and demands an efficient implementation. Ideally, the implementation must cope with the growth of users as well as the growth of the underlying database.

We here set out to improve on this last category in various aspects: we describe the design and implementation of a scalable, distributed architecture that can handle queries from multiple simultaneous users. Each user can provide multiple samples in form of BIOM tables [6], representing high-dimensional (but sparse) Operational Taxonomic Unit (OTU) abundance vectors as measured by 16S rRNA sequence counts. For comparability reasons, we require that all samples are derived from consistent closed reference OTU picking. These abundance vectors are not only compared with each other but are searched and contextualized against samples from a broad range of environments. We therefore strive to employ the most comprehensive database of microbial communities available. NCBI’s Sequence Read Archive (SRA, [7]) is likely to be the largest repository of 16S rRNA profiles. However, SRA usually stores raw sequence reads leaving further processing, especially quality control, to the users. Furthermore, the provision of additional metadata such as those specified in MIMARKS as well as barcodes, primer sequences are study-specific, not standardized and therefore difficult to automatize. Qiime-DB/Qiita [8] is a microbial study management platform, supporting multiple analytical pipelines. However as with SRA, it does not have the capability of querying a user-provided sample against the underlying database. Likewise, tools like VAMPS [9], myPhyloDB [10], Mothur [11] and Megan [12] can compare, store and analyze microbial community profiles, but do not provide a complete similarity search against a comprehensive database. We aim to complement those tools by providing such database search while still facilitating interoperability through standardized file formats such as BIOM and FASTA. This also includes the incorporation of the most commonly used phylogenetic and non-phylogenetic distance measures for microbial communities: weighted UniFrac and Bray-Curtis dissimilarity, respectively. Weighted UniFrac calculations are computationally expensive, and was previously tackled by using Trie-index based heuristics to reduce the number of comparisons [5]. We show that this approach is afflicted with a considerable number of False Negatives (i.e. very similar samples were overlooked due to slightly differing indices). To overcome this issue, we deploy an accurate, sublinear similarity search using Geometric Near-neighbor Access Trees (GNAT, [13]) which facilitate similarity searches in high dimensional metric spaces. In addition, we deploy AESA (Approximating and Eliminating Search Algorithm), [14], which excels in query-intensive systems, i.e., in situations where heavy precalculation is feasible and the number of distance calculations per query needs to be kept minimal. Thanks to the recent realization that Weighted UniFrac is a metric ([15]), we show that it is suitable for similarity searches in high dimensional metric spaces using GNATs and AESA. Finally, various aspects for microbial community comparison are taken into account: copy number correction ([16]) and rarefaction in order to deal with varying sequencing depths of samples.

## Implementation

To tackle the problem of increasing user-base and increasing popularity of sample querying systems, we present a web application called Visibiome. Visibiome features a distributed architecture to maximize usability and minimize dependency issues for personal and public deployments. In its entirety, Visibiome is developed using open-source software. The Visibiome core is built using the web development framework Django which has several benefits for distributed web application development (e.g. it is database agnostic and modular), which is fitting for computationally-heavy search query systems since single-machine implementations will not scale very well with multiple concurrent queries. Here, we explain the modularization of Visibiome and how it scales as a search engine.

Visibiome uses MySQL as the preferred relational database management system (RDBMS). MySQL is favourable for being open-source, well-received, able to handle complex relational models and is performant [17]. Visibiome is connected to two main databases: (i) the Visibiome database (*D*_*V*_) and (ii) the indexed microbiome database (*D*_*M*_). *D*_*V*_ contains user schema and user query metadata while *D*_*M*_ houses an annotated database assembled from various other microbiome databases (described in [18]), comprising additional information for samples (such as sample size, Environmental Ontology (EnvO) annotation) and GreenGenes OTUs (taxonomic lineage, 16S rRNA copy number). Visibiome mainly performs complex, multiple read queries on both databases and few, simple write queries on *D*_*V*_. While it can reduce connection lag to install *D*_*V*_ and *D*_*M*_ in the same vicinities as the computation server, competitions for CPU threads can happen when a query is invoked. Visibiome prefers decoupling the database from the server. This separation enables the web server to focus on serving the web application while a dedicated MySQL server performs complex queries.

Similarly, for the web server, CPU thread hogging of the query computations over the service of web pages can happen. In this scenario, it is likely that usability of the system will diminish. To remedy this, we deploy Celery for task queuing and deferring [19]. Celery enables multiple tasks to be processed in parallel provided that the server has enough CPUs to match the number of “workers” (entities which perform computations). Task queuing is automatically managed by Celery and can be configured to prioritize urgent tasks (for example, lengthy computations). Celery requires a message queuing service to queue the tasks. In Visibiome, we employed Redis as the message queuing service for its high-performance and speed [20].

**Fig. 1:**
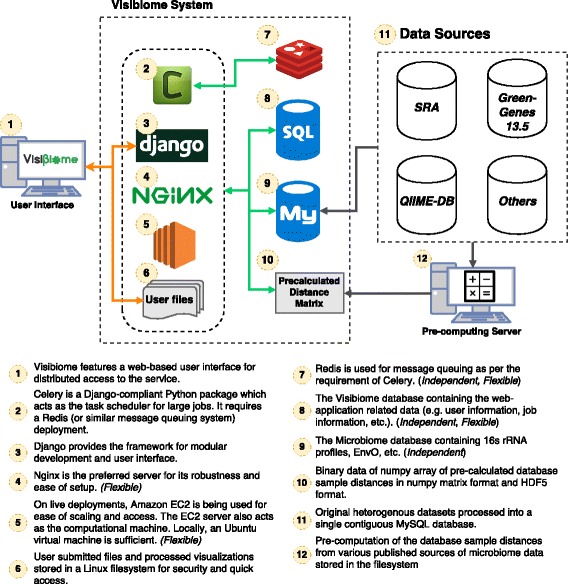
Visibiome’s schematic. A brief schematic of a typical Visibiome deployment showing implemented technology (depicted as different shapes and models) and data flow paths (depicted as *arrows*). Visibiome features a distributed architecture. Independent entities can be deployed as a dedicated service rather than coupled to the web server. Flexible entities can be customized to user preferences such as RDBMS. The schematic shows how data are transferred between the implemented technology. The *orange* paths depict user interaction to the web server. The *green* paths depict data flow when queries (computations) are performed. The *grey* path shows the set of original databases compiled into a single MySQL database which contains pre-computed sample distances and metadata

Newer standards of server technology has made deployment of web services highly automated. Legacy solutions involving configuration is being replaced by conventional means. Interfacing web services through Web Service Gateway Interface (WSGI) is currently a growing standard of which Visibiome takes advantage. Visibiome is served using Nginx and uWSGI to improve speed over traditional Apache servers. To ensure rapid content delivery, considerations have been made for transferring large files and potentially blocking code. For scalability, we deploy Visibiome on an Amazon AWS EC2 server featuring flexible CPU and memory scaling and providing global access for users. A typical schematic of the technology and data flow of the Visibiome system can be seen in Fig. 1.

### Using Visibiome and the user interface

Visibiome is free for public use through its web interface on https://visibiome.org/ (see for more options in “Availability” section). Before submitting a sample into Visibiome, users are encouraged to register an account. Anonymous submissions will be stored in a private guest account which is automatically created upon submission. It should be noted that although guest accounts are private, all guest accounts share the same password. Also, guest accounts are temporary and will be deleted within 24 h along with any submissions, uploaded files and processed files attached to the guest account. To avoid loss of processed submissions, the user can upgrade the guest account into a full-fledged account by updating their username and password for the guest account.

Submissions into Visibiome are OTU tables in BIOM format [6]. These can be produced with currently available services such as VAMPS [9] or Qiime [21]. The BIOM format is notably common (for marker-gene data), standardized and size-efficient. Visibiome accepts BIOM tables in the following file formats: TSV (tab separated values), JSON or HDF5 which allows the data to be human-readable and also space-efficient. User-submitted BIOM tables must be produced by closed-reference OTU picking against GreenGenes 13.5 [22] in order to ensure comparability to database samples, but also guarantee fast taxonomic composition analysis of user samples. Visibiome will yield errors for BIOM tables subjected to *de novo* and open referenced OTU picking. This restriction is imposed by the indexing of *D*_*M*_. Note that closed reference OTU picking is far more suitable for the type of database search presented here, and we further justify this choice in the “Results and discussion” section. In addition, we provide the possibility for users to submit FASTA files with sequence identifiers that are in a format as expected by QIIME’s OTU picking scripts (<sample-id>_<sequence-id>, see QIIME’s documentation on file formats, qiime.org/documentation/file_formats.html). Visibiome automatically recognizes FASTA files (by file extension) and picks OTUs compatible with the outlined workflow. For full metagenomic shotgun datasets we recommend to preprocess the sequences with tools that produce taxonomic profiles, such as SortMeRNA [23]. Last but not least, Visibiome works with normalized and non-normalized OTU counts by prompting users to normalize 16S copy numbers during query (which is achieved by extracting pre-calculated values for all OTUs from the database, populated with PICRUSt’s script normalize_by_copy_number.py [24]).

Present-era web applications often feature data management and browser-based user interface; for example, in the realm of bioinformatics: [5, 9, 25, 26] and many others. Considering the numerous combinations of query settings and outputs available in Visibiome, a simple but sophisticated organization of these information is imperative. We ease client-side file management by recording user submissions as individual entities called jobs. When performing a query, a user provides settings and filters for a job, along with the desired BIOM file, before submitting it into the system. All jobs are private to the submitting user and are conveniently listed in the user dashboard. Jobs are annotated with metadata which includes links to access the output visualizations, time-based information, all user-selected settings during query and any error messages encountered during processing. Jobs can also be removed and rerun.

**Fig. 2:**
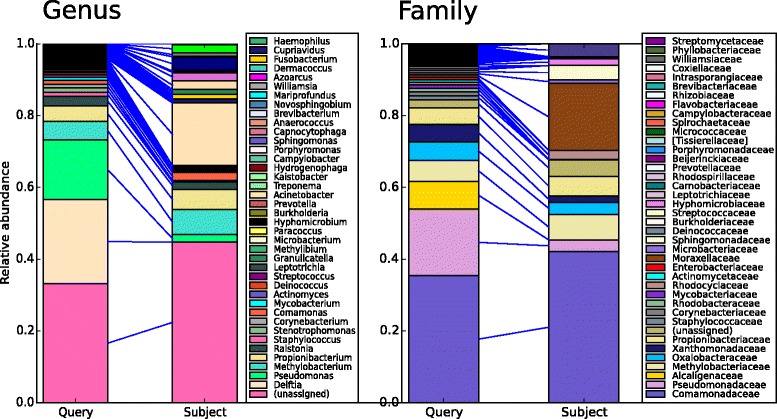
Compositional comparison of query sample and matched sample. The barcharts show compositional correspondences on genus-, family-, and phylum level. The fractions of constituents are consistently ordered with respect to the size in the query sample. This facilitates visual inspection as to why samples have been deemed similar in terms of the chosen distance measure

**Fig. 3:**
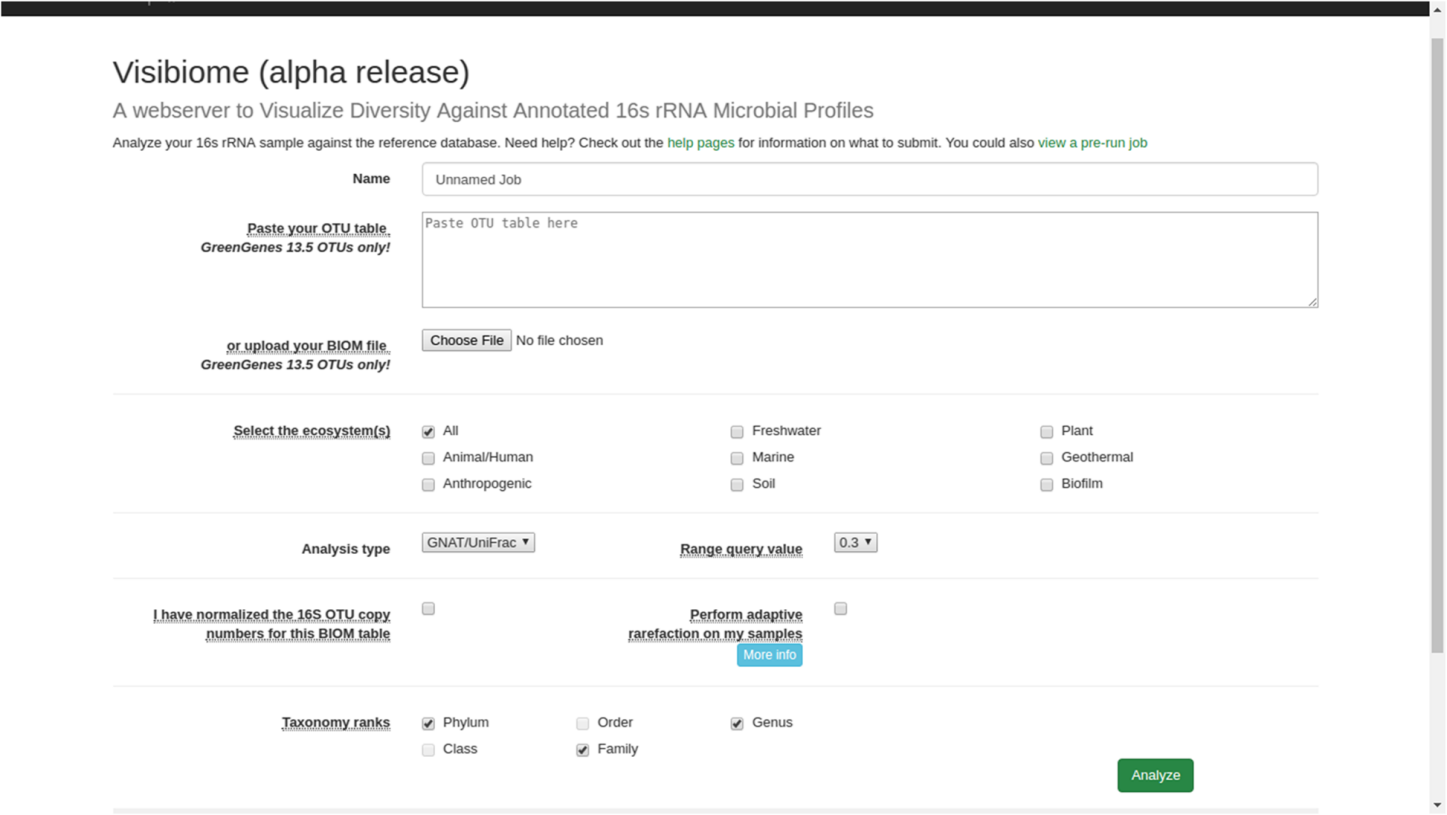
Screenshot of the user interface. User interface with input mask, providing the user with several ways to upload an OTU table in BIOM format or raw sequences in FASTA format and to select search criteria to narrow the search to a subset of predefined ecosystems. Users can also supply other available search parameters to a query such as the distance measure and the ranking levels for visualization

Visibiome produces visualizations of user queries as an output. Visualizations are displayed on the user’s browser by leveraging cutting-edge plotting libraries: matplotlib [27], d3.js [28] and mpld3 [29]. These output visualizations are separated into different pages. The “Ranking” page presents a high-level summary of the search query. Closest matching database samples to the user-queried samples are ranked into a list of cards. Each card contains metadata relating to the database-matched samples and, where possible, provide a URL to the source of the data. The “Ranking” page also features barcharts for comparison of sample compositions, thus allowing users to inspect the culprit of taxonomic similarity between query samples and matched samples, see Fig. 2. Visibiome produces interactive, zoomable barcharts for up to three user selected taxonomic ranks. An interactive, metadata-labelled, principle coordinate analysis (PCoA) plot is also available with zoom functionality to closely distinguish sample points. Queried samples can also be contextualized through a metadata-labelled dendrogram plot of the closest matches. More details regarding the contextualization of the samples can be found in later sections of this work. For a list of secreenshots of Visibiome, see Fig. 3.

#### Search algorithms

In order to speed up search against a large database, we deploy two fast search algorithms: Geometric Near-neighbor Access Trees (GNATs) [13] and the Approximating and Eliminating Search Algorithm (AESA) [30]. While GNATs are suitable for larger databases due to the lower (subquadratic) precalculation cost, AESA excels by reducing the number of distance (metric) computations per query to *O*(1) on average. We chose GNATs and AESA over other similarity search techniques due to their great performance in high-dimensional metric spaces. We combine both algorithms with an optimized weighted UniFrac calculation as metric. As we use GreenGenes 13.5 as closed reference, every sample is expressed as a sparse vector of (relative) abundances of dimensionality equal to the size of our OTU reference (99.325 OTUs for 97% sequence identity) which we denote as *L*.

We use the Python based GNAT implementation from coord_util [31], which is compatible with any user defined metric. We implemented AESA according to the algorithm description in [32]. We use our previously published and indexed MySQL database for rapid sample information retrieval [18]. We calculate the weighted UniFrac metric using an optimized version of EMDUnifrac [33], an efficient algorithm inspired by the recognition that weighted UniFrac is a metric equivalent to the Earth Mover Distance (EMD) [34]. EMDUnifrac starts with relative abundance differences at the leaves of the phylogeny and propagates “earth” (here: abundance differences) in a bottom-up manner, while balancing sources and sinks during each traversed node. The original algorithm traverses every node of the phylogeny and its complexity is provided with *O*(*L*). Note that the chosen choice of similarity threshold (here 97%) relates to *L* and hence affects the emdusparse In our case, *L* is very large. To further reduce the complexity, we base our optimization on the observation that most abundance vectors are sparse (i.e. 0 for most OTUs) and thus do not contribute to the distance calculation. We therefore consider only leaves that have non-zero abundance differences. To account for the varying depth of the GreenGenes phylogeny we perform tree traversal strictly level-wise using a list of dictionaries, one for each level. The dictionaries maintain the amount of unbalanced “earth” received from its children. Only when all children are processed can the remaining amount be propagated to the node’s parent, if the amount is non-zero. We refer to this algorithm as emdusparse.

**Fig. 4:**
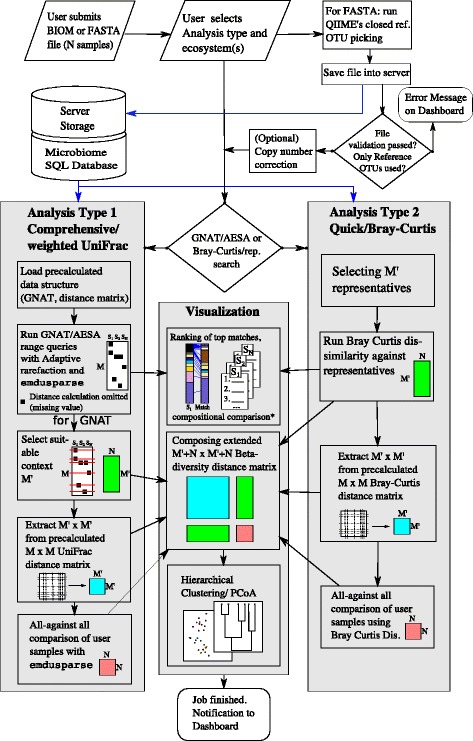
Visibiome’s workflow. The figure outlines the typical workflows when using Visibiome. The upper part deals with the Web interface and user interaction. At the core of Visibiome are two analysis types, comprehensive/phylogeny based and quick/non-phylogenetic distance based. Note that Analysis Type I (GNAT search) selectively compares to chosen database samples during GNAT traversal which are specific to the query sample. For some parts of the visualization however, a complete beta diversity distance matrix is required. As a consequence, the algorithm chooses M’ samples from the intersection of the individual search spaces. Moreover, barcharts for compositional comparisons ^∗^ are currently only generated for Analysis Type I

We build GNATs for the entire database comprising |*M*|=24.615 samples as well as for individual ecosystems. We denote the cardinality of the user-submitted samples as |*N*|, which varies between 1 and 10 in the interest of timely computation. Contextualization through principal coordinates analysis (denoted as PCoA) and Hierarchical Clustering (denoted as HC) requires a complete |*M* ^′^∪*N*|×|*M* ^′^∪*N*| distance matrix that includes meaningful samples from our database (*M* ^′^⊆*M*) as well as the provided user samples (*N*). For each user sample, we initiate a GNAT range search with a distance threshold of 0.3 (motivated by the empirical *p*-value discussed below and the amount of pruning that is possible with smaller thresholds). All computed distances of encountered comparisons are recorded; however, the encountered GNAT nodes for each search differ from user sample to user sample, in particular when user samples are very different from each other. In our implementation, a full beta-diversity distance matrix without missing values is required for contextualization (HC, PCoA). We therefore consider only those database samples that have been compared to all user samples during the individual GNAT searches. From this set, we retain only those that are within the top *k* (default 20) for at least one of the user samples, yielding a conveniently sized context *M* ^′^. Note that the encounters of samples associated with GNAT nodes make for a meaningful combination for contextualization: a few remote samples (from top-level GNAT nodes) and a number of more closely related samples as the GNAT search narrows in. This procedure yields a |*M* ^′^|×|*N*| distance matrix without missing values (see also Fig. 4, second and third item in the box for Analysis Type I). We then compose the complete matrix as follows: the |*M* ^′^|×|*M* ^′^| distance matrix is extracted from the pre-calculated |*M*|×|*M*| matrix (fourth item in Analysis Type I, Fig. 4). The required (|*M*|2)=302.961.420 weighted UniFrac calculations were performed on our in-house High Performance Computing Center using a parallelized script splitting the task into 10.000 jobs over 384 processors. In order to extract the submatrix from this matrix (4.6 GB on disk space), we use NumPy, Dask [35] (which facilitates out-of-core computation), and fancy indexing with the matrix being stored in HDF5 format. The user samples *N* are compared with each other, calling emdusparse for each pair (fifth item in Analysis Type I, Fig. 4). We finally combine all submatrices to obtain the complete beta-diversity distance matrix for all samples including the context *M* ^′^ and the user samples *N*.

Note that GNAT and AESA require distance measures that are metrics, i.e. fulfil the triangle inequality, are symmetric and non-negative, which is not the case for the popular Bray-Curtis dissimilarity. To address the lack of such properties, we introduce a coarse-level search algorithm by searching against up to 1000 randomly-selected representative samples (derived from HC) seeded from a pool of representatives by an ecosystem filter. Once completed, the user samples are contextualized against the representative samples by means of visualizations. We pre-calculated the Bray-Curtis dissimilarity for a large subset of 10.500 samples in the database. For PCoA/HC that requires a complete beta-diversity distance matrix, a query sample still would give rise to *M* individual comparisons. However, by comparing only against representatives, we can substantially reduce the amount of comparisons to identify the top *k* samples and to produce a relevant beta-diversity distance matrix.

#### Contextualization

The dataset used in this work to contextualize user-submitted samples is described in [18]. Notably all samples are associated with metadata. In particular, standardized, hierarchically structured descriptors about the sample’s environment are utilized: every sample from QIIME-DB contains up to three annotations from the Environmental Ontology (denoted as EnvO, [36]), namely environmental material, environmental feature and biome. Other samples in the dataset did not have EnvO annotations originally and were added retroactively using text mining as described in [18]. For improved comprehension of context, further grouping of EnvO annotations into high-level ecosystems (soil, human-associated, fresh water, marine, plant associated, etc.) were carried out exploiting the hierarchical nature of the ontology, the details of which are also provided in [18].

## Results and discussion

**Fig. 5:**
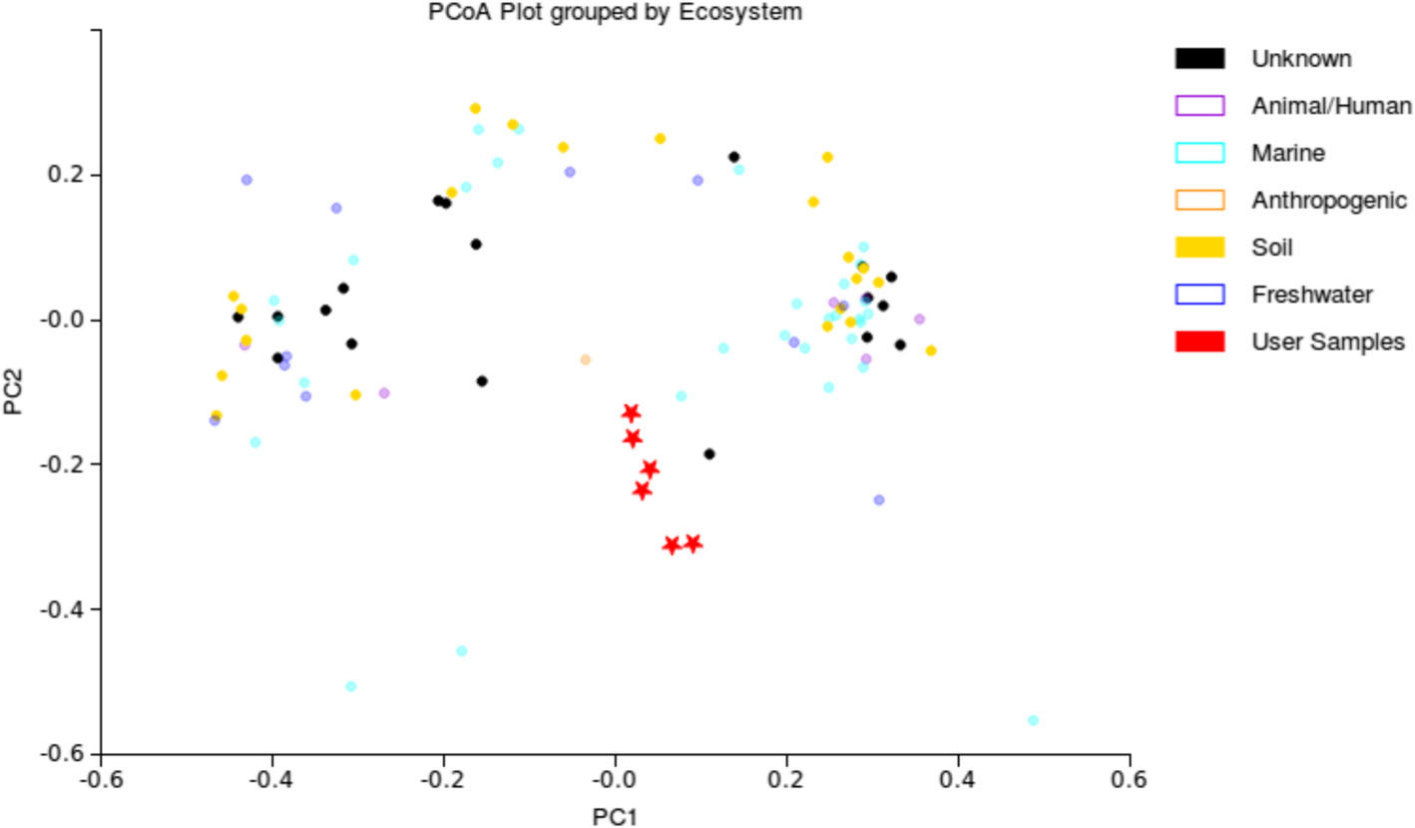
PCoA plot of user-submitted samples against closest matches. The figure shows a typical PCoA plot from the output of querying several samples (depicted as red star points) against the Visibiome database samples (depicted as circular points in varying colors). The PCoA plot allows users to contextualize the submitted samples against its closest matching database samples. Visibiome displays the matched samples with ecosystem labels and EnvO labels. Other metadata are also attached to each sample point (if available)

We here presented a multi-component architecture that performs search and contextualization of microbial community 16S rRNA profiles against a large database of samples from all environments. Several computational challenges are tackled. The overall work-flow is shown in Fig. 4. In summary, user samples uploaded to the web server undergo a series of analysis types, namely search against the database, yielding a ranking of closest matches. Subsequently, the algorithm constructs an extended distance matrix—while utilizing pre-calculated distances for database samples—in order to perform PCoA and HC of ranked database samples and user samples together. A typical result is shown in Fig. 5: the user can see the submitted samples in relation to each other *and* in the context of the closest matches. More screenshots are in the Additional file 1: Figures S4–S8. We provide two types of searches, one for the most popular non-phylogenetic distance measure (Bray Curtis dissimilarity) and one for the most popular phylogenetic distance measure, weighted UniFrac. The latter is a distance metric and as such lends itself to similarity search algorithms in metric spaces. The dimensionality of the metric space is in our case determined by the size of the deployed reference library, GreenGenes 13.5, as samples are represented as equal-sized OTU abundance vectors. The high dimensionality is thus a result of the recognized microbial diversity and it is conceivable that this number is to grow even further as more OTUs enter the reference. We reference [37], who reported 5.6 million OTUs from open reference picking.

### Feasibility of OTU picking strategies in online database search

While open reference or *de novo* OTU picking is desirable, it would incur further requirements and inaccuracies: in addition to extremely high dimensionality in open reference picking, OTU picking (at least for the de novo part) would be required for the entire database *after* user submission. Moreover, an all-encompassing phylogeny (including de novo OTUs) is needed to run UniFrac (or any other phylogenetic distance measure), a demanding feat best performed on full length sequences (it is not straightforward, how phylogenies for millions of OTUs should be generated). Last but not least, open reference/*de novo* OTU picking is not feasible for comparison of samples for which non-overlapping segments (i.e., different hypervariable regions where sequenced) which limits the scope of meta-analyses further. Instead, we here estimate the impact from the loss of information for the task of similarity search to show that closed reference based distances are a suitable approximation. We calculate *β*-diversity distances with and without sequences that don’t match the reference for a set of environmental samples that have around 66% matches against the reference (GreenGenes 13.5), see [18], Table S2 therein. The results show that distance calculations do not differ much (Additional file 2) and hence rarely affect the ranking in similarity searches.

### Search efficiency

**Fig. 6:**
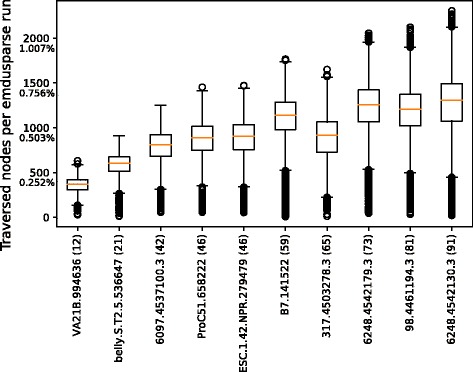
Efficient search through search spaces similarity search and sparse EMD-UniFrac (emdusparse). The number of nodes visited during an individual emdusparse traversal of the reference phylogeny reduces from 198.642 to an average of 400-1300 nodes, i.e. 0.2-0.6%, respectively. Note that for each boxplot we collected the traversal counts from all emdusparse comparisons during the entire GNAT search for the respective sample. The speedup is particularly noticeable for samples with few distinct or phylogenetically similar OTUs

We investigated the state-of-the-art Nearest Neighbor search techniques such as K-D trees, Ball Trees, and Vantage Point Trees explained in [32]. All of them performed poorly (i.e. resorted to brute force linear search) due to the very high dimensionality of the present search space. Only GNAT and AESA avoided a complete linear search, but the former still required several thousands of comparisons during a single query while the latter reduced comparisons significantly (for details, see Additional file 1: Figure S9. On the other hand, note that the pre-calculation of the complete |*M*|×|*M*| distance matrix constitutes the main computational challenge and is the central requirement for AESA. Therefore, contextualization and AESA search will only be possible for mid-size databases, while GNAT can go beyond. Since also the phylogeny-based distance measure calculation is computationally expensive, we not only minimized the number of calculations but also optimized the distance measure (weighted UniFrac) itself through building on recent results presented in [33], in which the authors present an algorithm that traverses the entire phylogeny (i.e., 198.642 nodes for the comprehensive GreenGenes phylogeny encompassing 99.325 OTUs from 97% sequences similarity clustering). The sparse vector based calculation presented here led to a reduction of traversed nodes as exemplified for ten samples in Fig. 6. The boxplot shows, for each sample, the number of traversed nodes of the reference phylogeny when emdusparse is invoked with the samples encountered during GNAT search (each yielding a data point, respectively). This approach requires only the traversal of subtrees above leaves with non-zero abundance differences. Thus, by traversing only the relevant part of the phylogeny, the number of visited nodes is roughly two orders of magnitudes smaller than the full-size phylogeny.

Note that rarefaction further decreases the number of non-zero entries in abundance vectors by ridding low abundance OTUs. Also note that traversal is generally faster for less complex samples with lower numbers of OTUs, i.e., lower (phylogenetic) *α*-diversity.

We empirically evaluated the running time of Analysis Type I and Analysis Type II by simulating user submissions. Each submission contains varying number of samples and are distributed randomly. For GNAT search and Bray-Curtis distance, the number of samples range from 1 to 10 samples; for AESA search, 10 to 100 samples in intervals of 10. Samples were randomly generated from various sources such as NCBI SRA, MgRAST and unpublished samples, meaning that submissions can contain samples which are very distant and possibly foreign to the server samples. To be conservative, we measured the running time of each analysis type from the moment the submitted BIOM file was validated. The preceding measurement takes into account all facets of the computations in Visibiome: computation of pairwise distances, querying of the pre-indexed database, queuing times and generation of visualization files.

The evaluation was done on a t2.medium AWS EC2 machine (specified to have 2 vCPUs and 4GB of RAM) utilizing two Celery workers to perform search queries. We subjected the submissions into two scenarios: (i) when the server is under no stress and search jobs are initiated infrequently and (ii) when the server is under stress of a large influx of jobs. We make our case by performing searches against the “All” criteria, implying searching over all ecosystem types, which is a heavy workload. To artificially replicate scenario A, a script automatically submits a new search job every 15 min. For scenario B, the time interval between new search jobs is 15 s. A total of 200 jobs were submitted split over 10 sample sizes giving 20 data points per sample size.

**Fig. 7:**
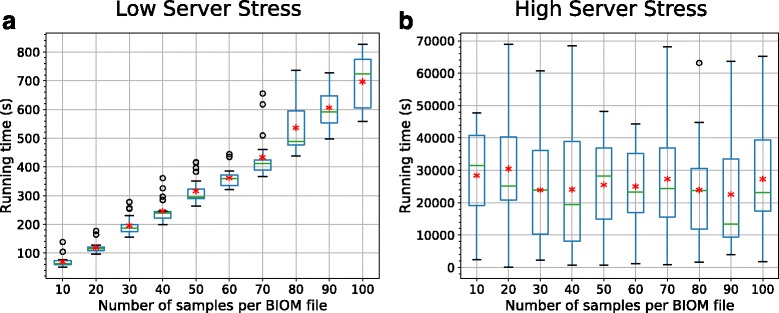
Boxplots of search query time for different number of samples in AESA search (Analysis Type I) under different stress levels. The boxplots show the running time (as in “job completion time”) for search queries of different input sizes in low server stress scenario, (**a**), and high server stress scenario, (**b**). The *red asterisk* represents the average running time, calculated over 20 running times, for each input size. The complexity of performing a search query can be directly inferred from (**a**) while in (**b**) this correlation is confounded by the addition of long queuing time. This trend is similarly seen in Analysis Type II but with lower running time than those of Analysis Type I

**Fig. 8:**
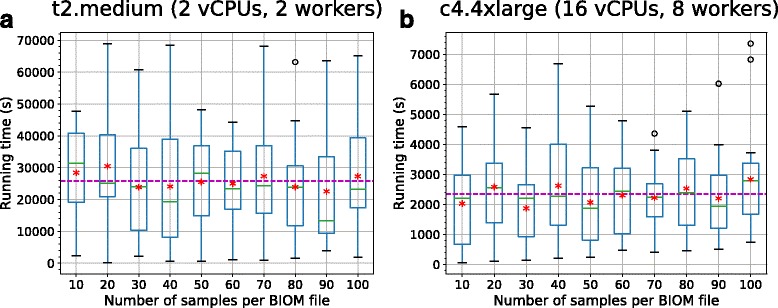
Boxplots of search query time for different number of samples in AESA search under high-stress scenario. The boxplots show the running time for search queries of different input sizes in high server stress scenarios on different AWS machine specifications. The *red asterisk* represents the average running time, calculated over 20 running times, for each sample size. The magenta dotted line represents the overall average time for the jobs (25815.3s in (**a**) and 2340.6s in (**b**)). It is clear that the system scales well provided more computations can be done in parallel

We found that in scenario A (Additional file 1: Figure S1(a)), Analysis Type II generally performs a search against “All” ecosystems in under one minute. This is attributed to the minimal queuing time for each search job and the coarse-grained nature of the Bray-Curtis analysis type. The processing time rises due to the complexity of pairwise distance calculations for increasing number of samples. The results of Analysis Type I (for both GNAT and AESA search) were similar: ranging from an average time of just under 2 min for a submission containing 1 sample (and 10 samples, respectively) up to 13 min for 10 samples (and 100 samples, respectively). See Additional file 1: Figure S2 and Fig. 7 for the empirical plots. For scenario B, it can be seen in Additional file 1: Figure S1(b) that, under heavy stress, Analysis Type II completes in around 5 min, on average. Again, similar trends were observed in Analysis Type I although queuing times were significantly longer (see Additional file 1: Figure S2 and Fig. 8).

This delay is due to the random queue into which jobs are put. Since jobs are collected asynchronously into a queue, and coupled with the speed at which jobs are invoked, jobs can be processed much later although requested earlier. The randomized queuing is unfortunately a feature of Celery which can possibly be mitigated by relaying jobs into priority queues. The algorithm for performing the relays are nontrivial and can have caveats in real scenarios due to randomness.

To evaluate the running time of range searches at different range values, we subjected a single sample size to the different meaningful ranges provided in Visibiome (which are 0.1, 0.2, 0.3 and 0.4). Similar to the tests we performed above, we executed 20 trials for each range with randomized samples under low and high stress. The results can be viewed in Additional file 1: Figure S10 and S11. As expected, we see similar trends to the analysis shown in Additional file 1: Figure S9 depicting a polynomial increase in number of comparisons. In high stress situations, the queuing of jobs levels the processing time although at 0.4 range the running time are mostly escalated.

It is important to note that the running time of search queries have been recorded to be as long as 48 h for AESA search (again, due to extended queuing instead of processing time) when the server is encumbered. We expect such scenarios to be unlikely and can be mitigated by scaling up the server specifications and employing more Celery workers. Note that thanks to cloud elasticity, this step has minimal requirements: we just had to order additional (virtual) hardware for a short period of time. To evaluate this, we scaled up the deployment server from t2.medium to c4.4xlarge with 16 vCPUS and 30 GB of RAM and employed only 8 Celery workers. We subjected the same high-stress scenario as previously described to GNAT and AESA search. The results for the simulation under high stress situations revealed that running times were significantly reduced by upscaling (see Fig. 8 and Additional file 1: Figure S3).

According to these findings, our suggestion for prospective heavy users is to download the prepared distribution of Visibiome and perform queries on their personal computers. We envision a way to make Visibiome more available to users: our modular, scalable architecture lends itself to crowd-deployed pool aggregation of Visibiome servers, from which users can select to quickly obtain results from their queries.

### Comparison to existing microbial community analysis tools/databases

Our web interface, job management and querying features are akin to those presented in IMNGS [25], although no searching of 16S rRNA profiles against a comprehensive database was provided in the system. Our approach compares well to Meta-Storms [5], the only other published microbiome search engine (to the best of our knowledge).

In the original work, Meta-Storms was described to require the building of a database from samples collected by the users before comparison is done. Visibiome features a comprehensive set of prepared samples against which user samples can be immediately compared removing the need to self-curate databases. While an example database was provided for demonstration purposes in Meta-Storms, Visibiome boasts a much larger sample database. The sample sources collected in Visibiome were formed from various study sizes but is broad in terms of ecosystems. Meta-Storms (as part of the Parallel-META pipeline [38]) can make use of the GPU for faster processing. On the other hand, Visibiome focuses on being catered to commodity server hardware, enabling cheap horizontal and vertical scaling. A summary of these differences are listed in Table 1.

**Table 1:** Comparison of key features between Meta-Storm and Visibiome

Criterion	Meta-Storms	Visibiome
Scalable architecture	No	Yes
Job-queuing/Scheduling	No	Yes
GPU support	Yes (Parallel-Meta)	No
Web interface	No	Yes
Database implementation	Indexed Flat files	MySQL and NumPy matrix
Database size	1,318 samples	24,615
Number of studies	18	2767
Input	Custom Preprocessing	FASTA or BIOM
Max. samples per submission	1	10 (GNAT)/100 (AESA)
Copy number correction	No	Yes
Adaptive Rarefaction	No	Yes
Interactive bar diagrams	No	Yes
PCoA	Yes (Parallel-Meta req.)	Yes
Hierarchical Clustering	Yes (Parallel-Meta req.)	Yes
Distance Measure	Unifrac-Like Score	EMD-UniFrac

Sample comparison in Meta-Storms is guided by indices derived from the ordered top five most abundant phyla of a sample. A quick analysis in our database of 24.615 samples shows a relatively large number of False Negatives, i.e., samples that would not be retrieved but should have been: from the (246152) pre-calculated distances, we chose distances that are below a certain threshold. From the selected distances, we check whether the corresponding sample pair has differing indices. The results are shown in Table 2. Even with a very small UniFrac distance of 0.1 (wrt. the utilized GreenGenes phylogeny), a substantial number (1402) of pairs of samples have differing top 5 phyla indices. With our Analysis Type I search we avoid this type of error all together. Instead we ensure efficient computation and 100% recall (wrt. to the user provided range search threshold) algorithmically: When using precalculated data structures, the search space is rapidly pruned by discarding all samples for which their representatives are too distant from the query, in terms of the chosen ecological distance metric.

**Table 2:** Observed False Negatives for top 5 phyla indexing (as done in Meta-Storms) in the presented database, i.e., the number of sample pairs below a specified threshold though with differing top 5 phyla indices; listed in dependence of various UniFrac thresholds

Weighted UniFrac	Total pairs	Pairs with	Percentage
threshold	below threshold	different keys	
0.05	28,247	351	1.24
0.10	96,977	1,402	1.45
0.20	846,107	14,993	1.77
0.30	4,847,874	91,902	1.90

Note that this type of error is avoided in our work by the use of GNAT data structures (Analysis Type I)

### Significance of matches

**Fig. 9:**
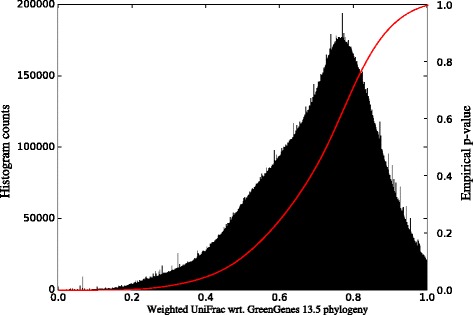
Empirical *p*-value calculation. The histogram shows the number of observed distances in bins of size 10^−4^ (*black bars, left y-axis*). From this we calculate the empirical *p*-value for a given distance as provided in Eq. 1 (*red line, right axis*)

We estimate the significance of a match *m* to a query sample *q* by calculating the empirical *p*-value (see Fig. 9): the computed distance between a query and a match is put into perspective by relating to all 302 Million observed distances, i.e., what fraction of them is smaller than the distance *d*(*m*,*q*), see Eq. 1.

1 $$ p = \frac{|\{m^{\prime}, m^{\prime\prime} \in M\ |\ d(m^{\prime}, m^{\prime\prime}) < d(m,q) \}|}{\genfrac{(}{)}{0pt}{}{|M|}{2}}   $$

In order to perform this computation efficiently, we pre-calculated a histogram of distances with 10.000 bins and in turn, the cumulative sum thereof, thus obtaining an accurate estimate for the numerator in Eq. 1.

### Application of Visibiome

Recall the findings in [1] where the authors found deep subsurface metagenomes to be similar to forest metagenomes. In light of this relatively “alien” sample, we took to Visibiome to discover other similar samples. We subjected the subsurface samples (which varied in the sampling depth) to the available search methods in Visibiome and discuss the results.

The output results from Visibiome can be viewed (publicly) at the following links

- GNAT search: https://visibiome.org/public/jobs/2801/details
- AESA search: https://visibiome.org/public/jobs/2800/details
- Bray-Curtis search: https://visibiome.org/public/jobs/2797/details

Surprisingly, the deepest sample (SRR1777625) exhibits similarities to database samples from entirely different environments, not reported previously. Figure 2 shows the compositional similarities to one of the closest matches, sample ID 4.1.CD.N from Qiita study 314: airborne microbial communities at high altitude. Both samples are composed of the families Comamonadaceae, Pseudomonadaceae, Methylobacteriaceae, Oxalobacteraceae, Xanthomonadaceae and Propionicateriaceae. On genus level, compositional similarities are less obvious. We argue that these nontrivial commonalities are rarely possible to retrieve manually from a search space of many thousands of samples.

### Availability

All described functionality is freely accessible through the web interface https://visibiome.org/. We provide the web interface generously but users may suffer from long queue times as a result of few CPUs available to process jobs in parallel. The choice of few processing CPUs is in the interest of minimizing hosting costs and it is encouraged that prospective users download a distribution of Visibiome for personal use. For those who intend to have a personal deployment of Visibiome, a VirtualBox distribution with Ubuntu and Visibiome is also freely available for download. The current implementation of Visibiome has some strict, albeit light, system requirements to be usable on an independent installation. Visibiome has only been tested to work as expected on Ubuntu 12.04 or newer. A minimum of 3 GB of RAM is recommended due to the need to load large files during computation; however, Dask based out-of-core computation enables functioning on lower specifications. Adequate storage is necessary for pre-calculated data, the indexed MySQL database and user-uploaded files. As explained previously, Visibiome pairs with Python libraries seamlessly giving users the freedom to customize and augment the computational scripts. The source code for Visibiome is available from Bitbucket licensed under GPL v3.0. The Git repository can be found at https://bitbucket.org/syaffers/visibiome.git and the authors welcome future contributors to the project.

## Conclusion

Visibiome is a microbiome search engine that boasts various architectural features to be scalable to many simultaneous user requests. It was demonstrated to serve computationally demanding jobs under high stress. We also showed that job completion time scales well through addition of more processors and according adjustment of number of workers. In addition to the state of the art job distribution and user management, users can provide multiple samples at once, which are then compared to each other as well as to the database.

We offer two types of analysis. The rationale for this is to provide one phylogeny-aware search technique with high accuracy (no false negatives as with phyla based indices) and one search with speed as top priority with a coarse-grained overview. For the former, we have implemented a search engine that is able to perform thousands of Weighted UniFrac calculations for a complete database search in a reasonable amount of time thanks to two main algorithmic advances: the use of GNAT and AESA structures for microbiome similarity search and the deployment of an optimized form of EMDUniFrac. Visibiome is available as a web server, as source code or as a pre-configured virtual machine.

## Availability and requirements

- **Project name:** Visibiome
- **Project home page:** https://bitbucket.org/syaffers/visibiome
- **Archived version:** Not applicable
- **Operating system(s):** Platform independent (tested on Ubuntu 12.04 and above)
- **Programming language:** Python
- **Other requirements:** Nginx 1.10, MySQL 5.5, Redis 3.0.6, Python 2.7+, QIIME 1.9.1, NumPy 1.10+, see requirements.txt in the repository for more Python package requirements
- **License:** GPL v3.0
- **Any restrictions to use by non-academics:** None
- The randomly generated BIOM tables used in this study are available in the Amazon AWS S3 bucket, https://s3.amazonaws.com/visibiome-data-files/supplementary/generated-biom.tar.gz, https://s3.amazonaws.com/visibiome-data-files/supplementary/AESA-biom.tar.gz
- Supplementary figures and data can be found in a git repository, https://bitbucket.org/syaffers/visibiome-supplementary/

## Additional files

Additional file 1 Supplementary figures are collected in this document. (PDF 723 kb)

Additional file 2 Spreadsheet comparing Open and Closed reference OTU picking The file is a spreadsheet in Microsoft Excel format. (XLSX 15 kb)

## Abbreviations

AESA: Approximating and eliminating search algorithm
EMD: Earth mover distance
GNAT: Geometric near-neighbor access tree
HC: Hierarchical clustering
OTU: Operational taxonomic unit
PCoA: Principal coordinates analysis
SRA: Sequence read archive
SRA: Sequence read archive
WSGI: Web service gateway interface
