# Supplementary material for: Visibiome: an efficient microbiome search engine based on a scalable, distributed architecture
Source: BMC Bioinformatics. 2017 Jul 24;18:353. doi: 10.1186/s12859-017-1763-0 (PMC5525214; doi:10.1186/s12859-017-1763-0)
Supplement: Supplementary file 1 — Supplementary figures are collected in this document. (PDF 723 kb) [file 12859_2017_1763_MOESM1_ESM.pdf]

Visibiome Supplementary Figures

Syafiq Kamarul Azman, Andreas Henschel, Zohaib Anwar

July 2017

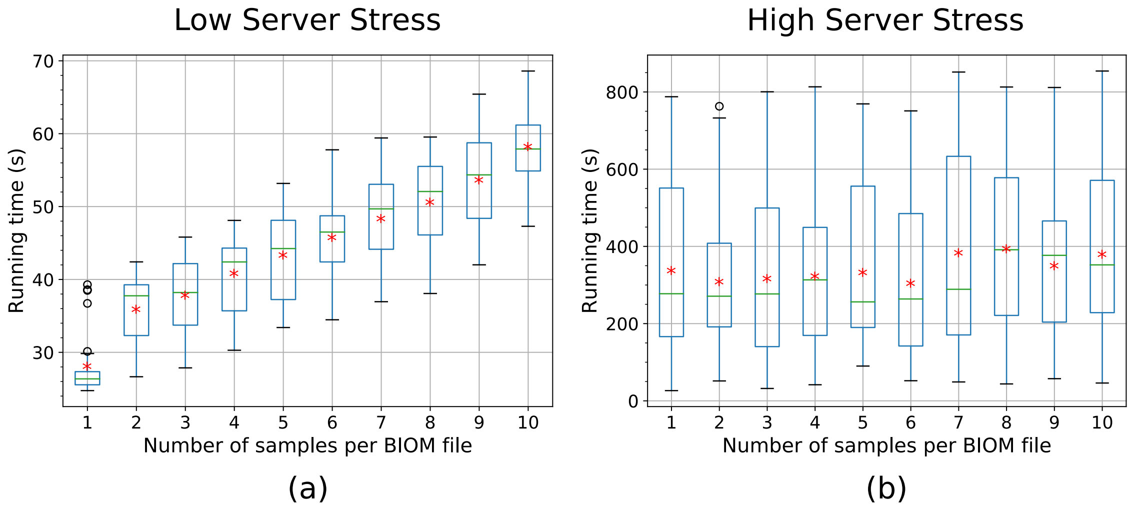

Figure 1: Barplot of Bray-Curtis analysis running time with different sample sizes in low and high stress situation on an EC2 `t2.medium` machine.

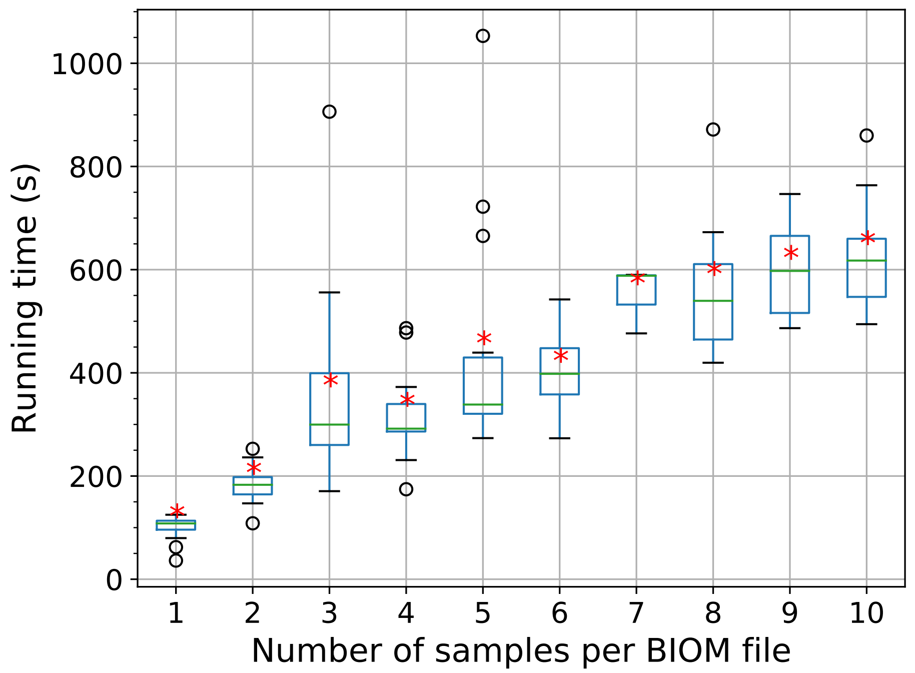

Figure 2: Barplot of GNAT/UniFrac running time with different sample sizes in low stress situation on EC2 `t2.medium` machine.

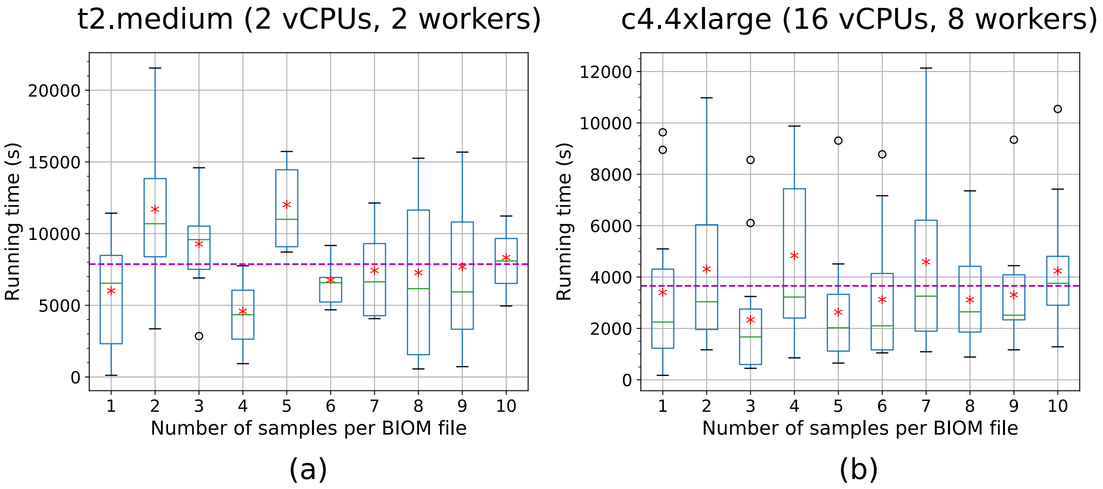

Figure 3: Barplot of GNAT/UniFrac running time with different sample sizes in high stress stiuations on EC2 `t2.medium` and `c4.xlarge` machines. The purple dotted line shows the average running time for all jobs.

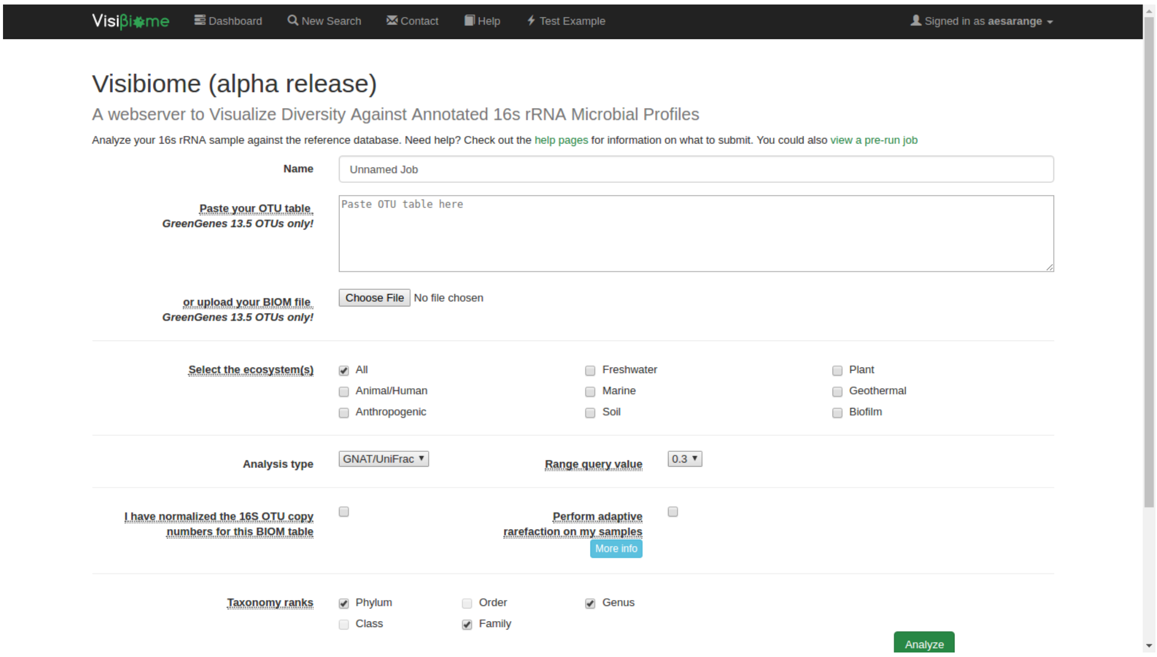

Figure 4: Screenshot of the search form for Visibiome with all combinations of settings for the search.

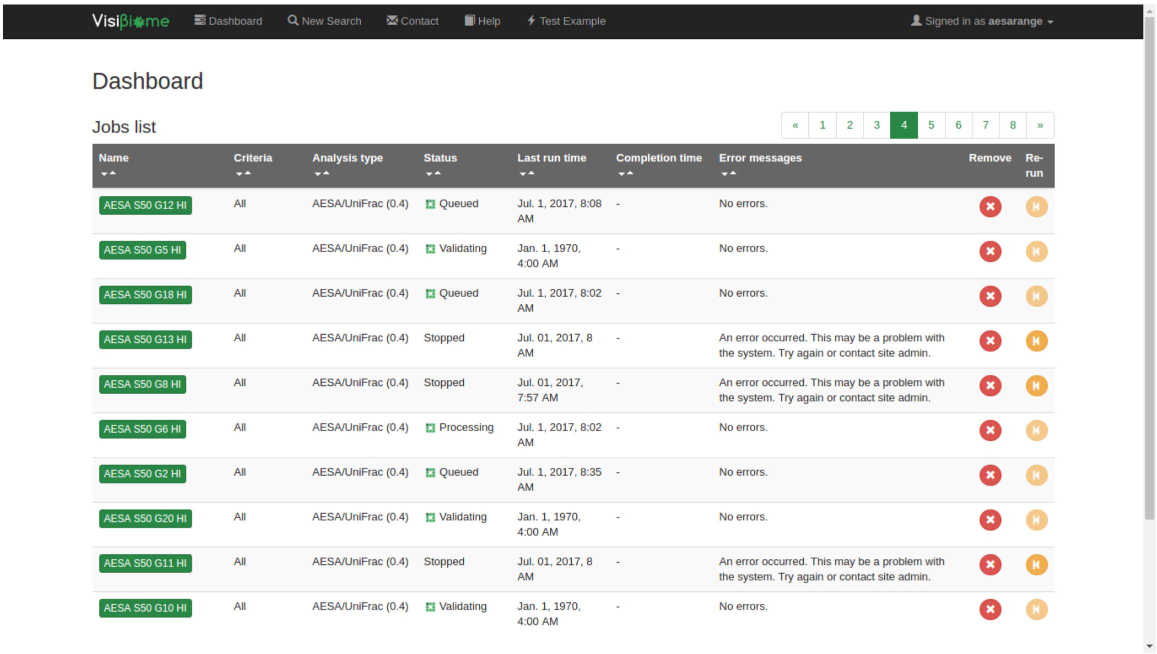

Figure 5: Screenshot of an example Dashboard for a user. The dashboard shows the running jobs and queued jobs as well as completed jobs and jobs that reached an error while processing. It features pagination, rerun and deleting.

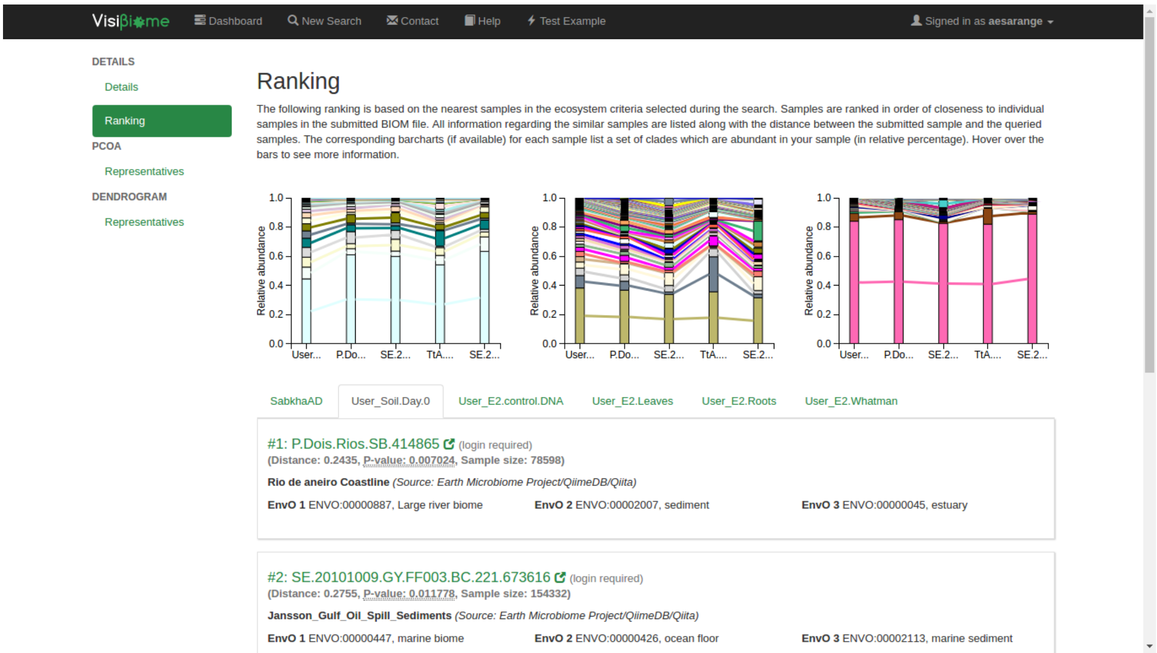

Figure 6: Screenshot of the barcharts visualization of a job. Barcharts are zoomable which enables users to delve into the culprits of the matches for their sample against the database samples.

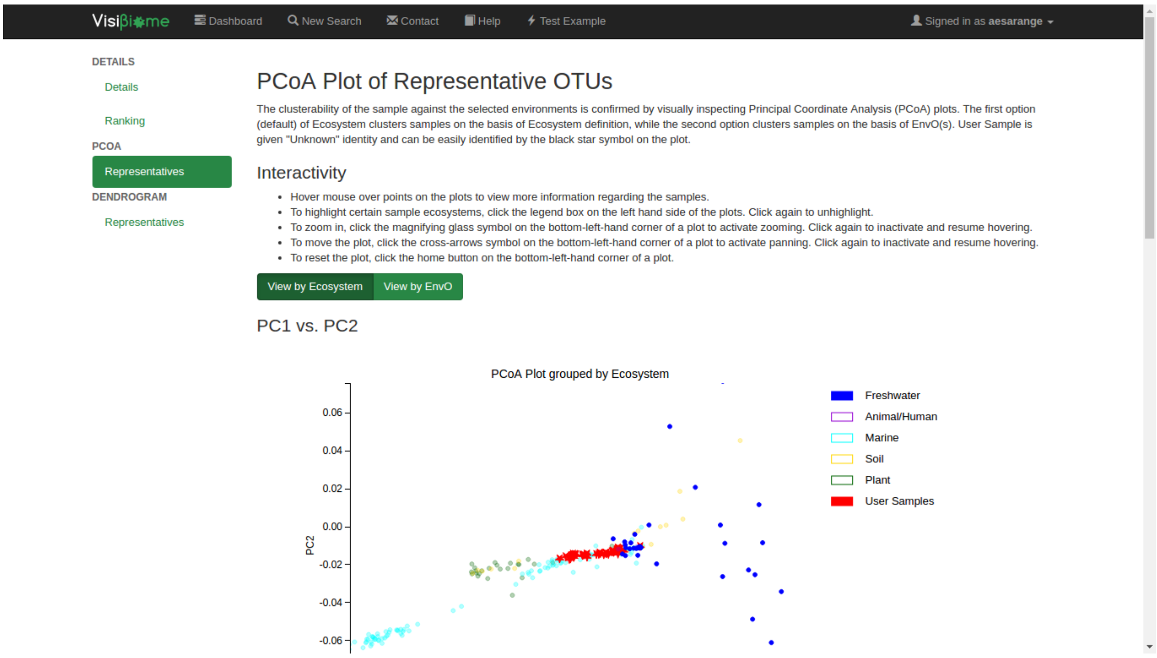

Figure 7: Screenshot of the PCoA visualization of a job. PCoA plots are 2-dimensional but the first three dimensions are enumerated. Plots are zoomable and hoverable to display more information for matched samples.

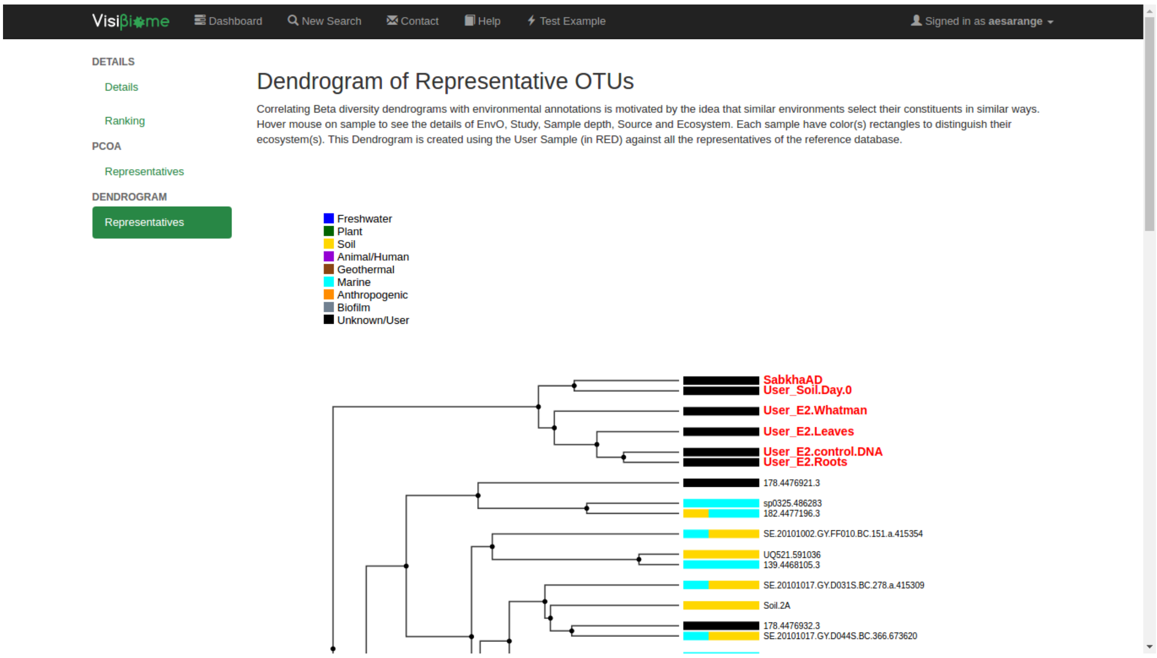

Figure 8: Screenshot of the dendrogram visualization of a job. Dendrogram plots show the relation between samples and the database sample's EnvO color. The samples can be hovered over to display more information.

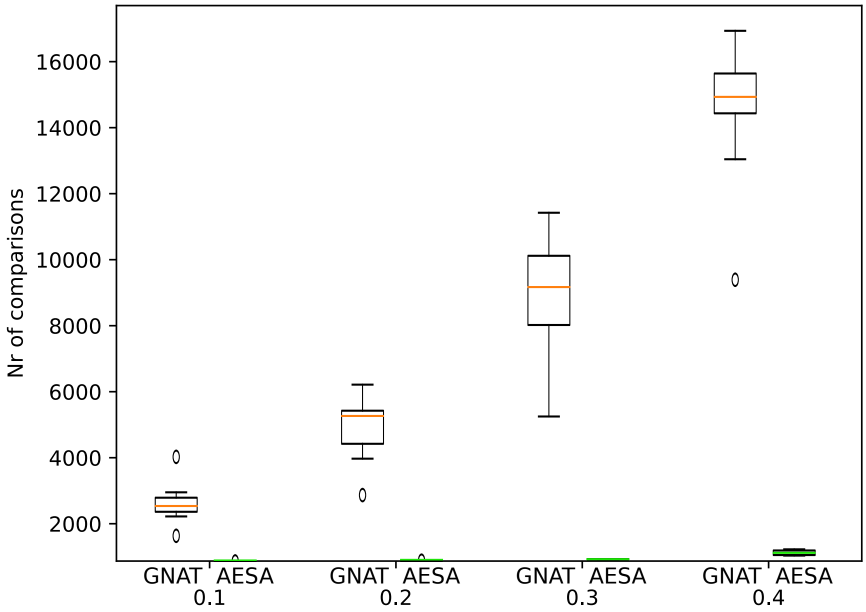

Figure 9: Barplot of the number of comparisons made for GNAT and AESA search on different ranges.

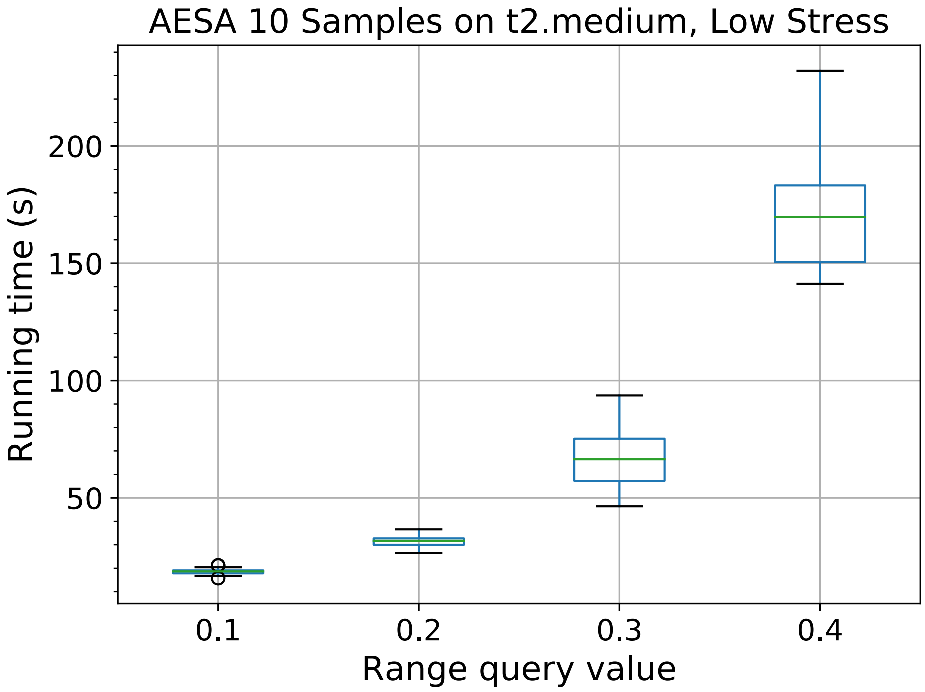

Figure 10: Barplot of AESA/UniFrac running time with different ranges in low stress situation on EC2 ‘t2.medium‘ machine.

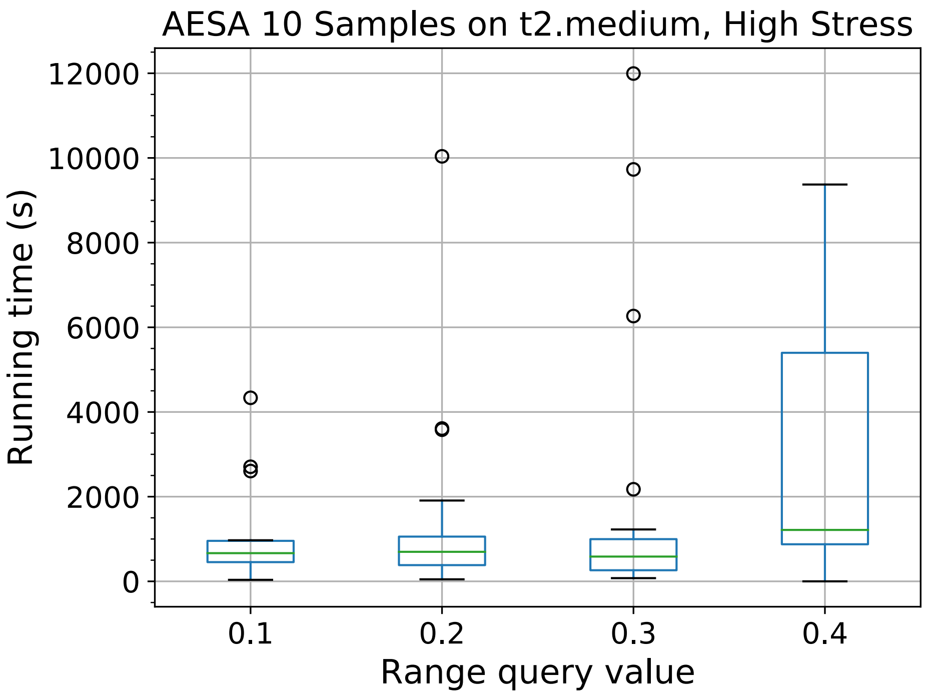

Figure 11: Barplot of AESA/UniFrac running time with different ranges in high stress situation on EC2 ‘t2.medium‘ machine.
